# Storage-Dependent Changes in Microplastic-Associated Recoverable Residues in Yogurt Containing *Bifidobacterium longum* subsp. *infantis*

**DOI:** 10.3390/toxics14060535

**Published:** 2026-06-20

**Authors:** Yasin Akkemik, Sedat Özcan, Veysel Doğan, Sedat Gökmen, Enis Fuat Tüfekci, Salih Erat

**Affiliations:** 1 Department of Food Hygiene and Technology, Faculty of Veterinary Medicine, Kastamonu University, Kastamonu 37100, Türkiye; yakkemik@kastamonu.edu.tr; 2 Department of Veterinary, Menemen Vocational School, Izmir Bakircay University, Izmir 35660, Türkiye; salih.erat@bakircay.edu.tr; 3 Department of Animal Nutrition and Nutritional Diseases, Faculty of Veterinary Medicine, Kastamonu University, Kastamonu 37100, Türkiye; vdogan@kastamonu.edu.tr; 4 Department of Veterinary Pharmacology, Faculty of Veterinary Medicine, Kastamonu University, Kastamonu 37100, Türkiye; sgokmen@kastamonu.edu.tr; 5 Department of Basic Medical Sciences, Faculty of Medicine, Kastamonu University, Kastamonu 37100, Türkiye; etufekci@kastamonu.edu.tr

**Keywords:** microplastics, yogurt, *Bifidobacterium longum* subsp. *infantis*, probiotic–microplastic interactions, Py-GC/MS, recoverable residue, dairy food safety, exploratory study

## Abstract

Microplastics (MPs) are increasingly detected in dairy products, raising food-safety concerns. Their behavior in complex food matrices and interactions with probiotic microorganisms remain poorly understood. This exploratory study evaluated storage-dependent changes in operationally defined, digestion-resistant recoverable residues in yogurt containing *Bifidobacterium longum* subsp. *infantis* (ATCC 15697). Yogurt samples were prepared with polypropylene (PP), polyethylene (PE), and polystyrene (PS), individually and in combination, and analyzed over 21 days of refrigerated storage. Gravimetric values served as relative, operational indicators of recoverable residues—not validated absolute polymer masses—while polymer identity was qualitatively confirmed by pyrolysis–gas chromatography/mass spectrometry (Py-GC/MS). *B. longum* subsp. *infantis* remained viable throughout storage (6.3–8.2 log_10_ CFU/g). All MP-containing groups showed consistent storage-associated decreases in recoverable residue fractions, greatest in PP, followed by PE and PS; probiotic-free controls remained stable. Polymer-specific Py-GC/MS signals were detectable at all time points. Because polymer identity was retained and the workflow was not validated for absolute recovery, findings are interpreted as storage-associated changes in extractability, filterability, and/or residue recovery—not as polymer degradation, mineralization, or biological removal. These in vitro observations are limited to the yogurt matrix and do not support extrapolation to livestock exposure, human dietary risk, or farm-to-fork transfer. Within these limits, the findings provide a preliminary, hypothesis-generating perspective on probiotic–microplastic interactions in fermented dairy products.

## 1. Introduction

Global plastic production has increased dramatically over the past century, reaching approximately 350 million tonnes annually, with projections suggesting that it may exceed 1.1 billion tonnes by 2050 [1]. Therefore, MPs, defined as particles smaller than 5 mm, have emerged as pervasive contaminants across environmental compartments, including soil, freshwater, marine ecosystems, and food systems [2,3].

Within the food chain, MP contamination has been reported in tap water, seafood, salt, honey, and increasingly in dairy products [4,5]. Yogurt, one of the most widely consumed fermented dairy products worldwide, is of particular concern in this context. Recent studies have identified polystyrene (PS), polypropylene (PP), and polyethylene (PE)—polymers widely used in yogurt packaging—as predominant plastic polymers detected in commercial yogurt samples [6,7]. These contaminants may originate from direct migration from packaging materials, as well as from contamination during processing and handling [4,8]. Dairy products are frequently exposed to plastic-containing materials throughout the production, processing, packaging, and storage stages, which may contribute to plastic-particle contamination of the final product [9,10,11]. Moreover, the high consumption rates of fermented dairy products such as yogurt increase their relevance in food-contamination and dietary-exposure assessments.

Following ingestion, MPs have been shown to cross the gastrointestinal barrier, accumulate in organs such as the liver and spleen, and induce adverse biological effects, including oxidative stress, inflammatory responses, DNA damage, endocrine disruption, and alterations in gut microbiota composition [12,13]. Similar concerns extend to food-producing animals: MP exposure has been shown to disrupt rumen microbiota composition and fermentation activity in cattle, impairing fiber digestibility and metabolizable energy yield, with downstream consequences for animal productivity and product quality [14,15]. In poultry, microplastic exposure has been associated with reduced gut microbiota diversity and intestinal integrity [16], highlighting that MP–microbiota interactions in livestock represent an underexplored but critical dimension of food safety. Given the high consumption frequency of dairy products across all age groups, the presence of MPs in yogurt raises significant food safety concerns.

Probiotic microorganisms are known to interact with a wide range of chemical contaminants, including mycotoxins, polycyclic aromatic hydrocarbons (PAHs), and organochlorine pesticides, through mechanisms such as binding, biotransformation, and detoxification [17,18,19]. These interactions suggest that probiotics may also influence the behavior of emerging contaminants such as MPs within food systems. *Bifidobacterium longum* subsp. *infantis* (formerly *Bifidobacterium infantis*; reclassified by Mattarelli et al., 2008 [20]), a well-characterized member of the infant gut microbiota, exhibits notable metabolic versatility and the ability to produce bioactive compounds with antimutagenic and detoxifying properties [21,22]. The rationale for the present study rests primarily on the well-documented capacity of probiotic bacteria to bind and biotransform diverse organic contaminants [17,18,19], together with the known surface-adsorption interactions between lactic acid bacteria and plastic particles [23,24]. In addition, a single preliminary study has reported that *B. longum* subsp. *infantis* may utilize PP as a carbon source under aerobic conditions [25]; however, this report has not been independently replicated and contains an internal inconsistency (an organism described as anaerobic studied under aerobic conditions). It is therefore not treated as mechanistic support but is noted only as a hypothesis-generating observation, and the present hypothesis is regarded as exploratory.

The One Health concept recognizes the interconnectedness of environmental, animal, and human health and provides a useful background framework for considering contaminants that may move across ecosystems and food chains [26,27]. In the present study, however, this framework is used only to motivate the broader food-safety relevance of MP behavior in dairy matrices; the experimental design itself is limited to an in vitro yogurt model and does not allow direct conclusions regarding animal exposure, human health outcomes, or farm-to-fork transfer.

Despite these promising findings, the behavior of MPs within real food matrices in the presence of probiotic microorganisms remains insufficiently explored. To date, only limited studies have addressed time-dependent changes in recoverable MP fractions in complex fermented dairy systems under realistic storage conditions. Although gravimetric and Py-GC/MS approaches are individually well established in environmental microplastic research [28,29,30,31], their combined application to a probiotic-containing fermented dairy system under realistic storage conditions remains uncommon.

We hypothesize that the presence of metabolically active *B. longum* subsp. *infantis* is associated with measurable, time-dependent changes in the operationally recoverable MP-associated residue fraction within a yogurt matrix during refrigerated storage. This hypothesis is exploratory and does not presuppose a specific underlying mechanism such as biodegradation. The present study therefore investigates storage-dependent changes in the recoverable residue fractions associated with PP, PE, and PS MPs in a yogurt matrix containing *B. longum* subsp. *infantis* while simultaneously evaluating probiotic viability and polymer-specific qualitative signal behavior using Py-GC/MS. Although a broader One Health perspective motivates the background, the study objectives are limited to the in vitro yogurt matrix; extrapolation to livestock-associated transfer, human dietary intake, or health risk is beyond the scope of the experimental design.

## 2. Materials and Methods

### 2.1. Microorganisms and Plastics

*Bifidobacterium longum* subsp. *infantis* (ATCC 15697) was obtained from the American Type Culture Collection (ATCC, Manassas, VA, USA). The cultures were prepared on *Bifidobacterium* Agar and in *Bifidobacterium* Broth (HiMedia Laboratories, Mumbai, India) under anaerobic conditions at 37 °C for 48–72 h. Before each yogurt production batch, broth cultures were harvested by centrifugation at 4000× *g* for 5 min. The resulting cell pellet was washed twice with pre-reduced PBS containing 0.1% cysteine and resuspended to obtain a target inoculum concentration of 2 × 10^9^ CFU/mL using a standardized dilution procedure. The final inoculum concentration was retrospectively confirmed by viable cell counting.

Polypropylene (PP; CAS 9003-07-0), polyethylene (PE; CAS 9002-88-4), and polystyrene (PS; CAS 9003-53-6) plastic powders were purchased from Sigma-Aldrich (Steinheim, Germany). Because particle-size distribution, morphology, and nano-sized fractions were not independently characterized in the present work, the materials are referred to conservatively as microplastics (MPs) throughout the manuscript. Commercial starter cultures containing *Streptococcus thermophilus* and *Lactobacillus delbrueckii* subsp. *bulgaricus* were obtained from a local dairy supplier.

### 2.2. Yogurt Production and Experimental Design

Raw bovine milk was collected from a single large lot from a small-scale dairy farm in glass bottles under cold-chain conditions and used within 24 h. The bulk milk was clarified through a muslin cloth and then divided into 100 mL aliquots in individual glass containers, with one per experimental group. Each 100 mL experimental unit was supplemented with the designated MP quantity (50 mg per 100 mL unit, equivalent to approximately 500 mg/L or 500 mg/kg yogurt) prior to pasteurization (80–85 °C, 20 min). For gravimetric monitoring, 1 g yogurt aliquots were processed, and the measured digestion-resistant residue was subsequently expressed on a normalized 100 g yogurt-equivalent basis to match the nominal 50 mg/100 g dosing scale. Therefore, the gravimetric values reported in the results should not be read as the mass recovered directly from a 1 g aliquot; they represent normalized operational residue values. The recovered residue includes all digestion-resistant material and is not equivalent to validated polymer mass. Residual proteins, lipids, salts, casein-derived aggregates, and other matrix-derived material may contribute to the residue, as characterized by the polymer-free matrix control included in the experimental design. After cooling to 43–45 °C, starter cultures (2% *v*/*v*) and *B. longum* subsp. *infantis* were added simultaneously. Yogurts were incubated at 41–43 °C for 3–4 h, cooled to 10 °C, and stored refrigerated at 4 °C.

Eight experimental groups were produced, each in three independent production batches: a polymer-free matrix control (K) and seven MP-containing groups (PE, PP, PS, PP + PE, PS + PE, PS + PP, and PS + PP + PE). For each group and sampling day, the three production batches were pooled prior to extraction to yield a single representative composite sample, which was then analyzed in triplicate by independent Py-GC/MS injections. Reported replication (*n* = 3) therefore refers to analytical injection-level replicates of the pooled sample, while biological variability between production batches was not separately resolved. The probiotic-free MP-only controls used in the gravimetric dataset are distinct from the polymer-free matrix control used for background characterization. All procedures were conducted in a laminar flow cabinet to minimize airborne contamination. Glassware was rinsed with MP-free ultrapure water before use. Personnel wore cotton laboratory coats and nitrile gloves throughout.

### 2.3. Probiotic Viability Determination

Viability of *B. longum* subsp. *infantis* was assessed on Days 1, 7, 14, and 21 of storage. Ten grams of yogurt was diluted in 90 mL Maximum Recovery Diluent and subjected to serial 10-fold dilutions (10^−1^ to 10^−6^). Aliquots of 1 mL from each dilution were plated onto *Bifidobacterium* Selective Agar Base supplemented with Bifido Selective Supplement A (FD250, HiMedia Laboratories, Mumbai, India) by the spread-plate method. Plates were incubated anaerobically (Anaerocult A, Merck, Darmstadt, Germany) at 37 °C for 72 ± 3 h. Characteristic cream-colored colonies were counted and results expressed as log_10_ CFU/g.

### 2.4. MP Extraction from Yogurt

MP extraction was based on the protocol of Leslie et al. (2022) [32], originally developed for low-protein, low-lipid biological fluids (human blood). The core enzymatic-digestion steps were retained (400 mM TRIS-HCl/0.5% SDS buffer, Proteinase K, 50 mM CaCl_2_, 50 °C digestion). To address the substantially higher protein, lipid, and calcium (casein micelle) load of the dairy matrix, an additional oxidative clean-up (H_2_O_2_/H_2_SO_4_) followed by TMAH derivatization—not part of the original blood protocol—was incorporated to remove residual organic matrix material prior to Py-GC/MS. Briefly, 1 g yogurt was diluted in 1 mL distilled water and homogenized. Fifteen mL TRIS-HCl buffer (400 mM, pH 8.0, 0.5% SDS) was added, and samples were heated at 60 °C for 1 h. Proteinase K (100 μL) and 50 mM CaCl_2_ (1 mL) were added sequentially, followed by incubation at 50 °C for 2 h, orbital shaking at 450 rpm for 20 min, and a final heating step at 60 °C for 20 min. Samples were vacuum-filtered through stainless steel membrane filters, oxidized with 10 mL H_2_O_2_ (30%) and two successive 0.5 mL H_2_SO_4_ washes, rinsed with 15 mL ultrapure water, treated with 10 μL tetramethylammonium hydroxide (TMAH), and dried at 40 °C prior to Py-GC/MS analysis. The oxidative step (H_2_O_2_/H_2_SO_4_) was applied to remove residual organic matrix material. Such oxidative treatments are known to potentially modify certain polymers, particularly polystyrene [33]; however, this step was applied identically to all groups and at all time points, so any such effect represents a constant systematic factor and cannot account for the polymer-dependent or time-dependent differences observed. Its possible contribution is nonetheless addressed in the Discussion and Limitations. Because the yogurt matrix itself changes during storage, the possibility that time-dependent matrix composition altered extraction efficiency, filterability, or residue formation cannot be excluded; this limitation is considered in the Discussion and Conclusions.

### 2.5. Py-GC/MS Analysis

Polymer characterization was performed on a Shimadzu GCMS-QP2010 Ultra gas chromatograph coupled to a Frontier Lab Single Shot Pyrolyzer PY-2020iS (Necmettin Erbakan University, Science and Technology Research and Application Center, Necmettin Erbakan University, Konya, Türkiye). Pyrolysis was performed at 700 °C, consistent with the established protocol for simultaneous identification and quantification of PE, PP, and PS by Py-GC/MS [28,29]. This temperature was selected to ensure complete thermal depolymerization of all three target polymers and to maintain comparability with the reference methods used for standard preparation and ion selection in the present study. Polymer standards (PE, PP, PS, PVC) were prepared in 1,2,4-trichlorobenzene (TCB) containing 0.0015% butylated hydroxytoluene (BHT) at concentrations of 5, 10, 20, 50, and 100 μg/mL as described by Steinmetz et al. (2020) [28]. Calibration of the three target polymers over the 5–100 µg/mL standard range yielded linear instrument responses (R^2^ > 0.99 for PE, PP, and PS), confirming the suitability of the diagnostic ions and pyrolysis conditions for reliable polymer identification. However, in the absence of an internal standard and matrix-matched recovery validation, these solvent-based calibrations were used to support polymer identity and method performance only, and not for absolute quantification of polymer mass in the yogurt matrix. Total ion chromatograms (TICs) were complemented by polymer-targeted extracted-ion chromatograms (EICs): PE (*m*/*z* 83, 97, 111), PP (*m*/*z* 70, 83, 126), and PS (*m*/*z* 51, 78, 104), following [29]. Each analytical run included three quality-control samples run alongside the experimental specimens: (i) a procedural blank (all reagents and consumables, no yogurt matrix), used to identify contamination arising from the extraction procedure itself; (ii) a polymer-free matrix control (yogurt processed identically to MP-containing groups but without added polymers), used to characterize background matrix contributions to the TIC/EIC signals; and (iii) a spiked recovery control for each target polymer (PE, PP, and PS individually spiked into polymer-free yogurt matrix at the nominal dose prior to extraction), used to confirm that polymer-specific EIC signals remain detectable after the full digestion and filtration protocol. The spiked recovery controls confirmed qualitative detectability of polymer-specific EIC signals in the yogurt matrix for all three polymers. Because no internal standard was employed, these controls support qualitative polymer identity confirmation but do not enable absolute quantification of extraction recovery efficiency.

The Py-GC/MS analysis in this study did not employ an internal standard and therefore can only confirm the qualitative presence of characteristic pyrolysis products of the target MPs, without achieving absolute or semi-quantitative quantification. The lack of internal-standard calibration makes it impossible to determine whether any apparent variation in EIC signal arises from actual fluctuations in MP mass concentration, dynamic matrix effects, extraction variability, or instrument response drift. Consequently, chromatographic signal intensities are not used for temporal quantification; chromatographic data are interpreted solely as qualitative presence/absence evidence for polymer identity. Future studies should employ internal standards such as poly(styrene-d5) or poly(4-fluorostyrene) [34,35] to enable absolute quantitative analysis and to validate the exploratory findings reported here.

### 2.6. Statistical Analysis

All analyses were performed on a single pooled composite sample per experimental group (combining three independent production batches), each measured in triplicate by independent Py-GC/MS injections (*n* = 3 analytical replicates per group). The reported variability therefore reflects analytical (instrumental) repeatability rather than biological (between-batch) variance, and all inferential outputs are interpreted accordingly. Because the reported standard errors derive from replicate injections of the same pooled extracted sample, they characterize instrumental repeatability and are expectedly small; they do not capture the larger variability that would arise from independent sample preparation, multi-stage extraction, and oxidative digestion across separate batches. The low standard errors should therefore not be interpreted as evidence of high experimental precision at the level of the full analytical workflow, nor as evidence of biological reproducibility. Data are presented as mean ± standard error (SE). The experimental design included storage time (days 1, 7, 14, and 21) as the within-group repeated factor, and treatment/polymer formulation (PE, PP, PS, PP + PE, PS + PE, PS + PP, and PS + PP + PE) as the grouping factor. In the operationally recoverable MP-associated residue fraction dataset (Table 1), the ‘Control’ groups contained the corresponding microplastics but no *B. longum* subsp. *infantis*, serving as the probiotic-free comparator; these are distinct from the polymer-free matrix control used for Py-GC/MS background characterization (Section 2.5).

Statistical analyses were performed using IBM SPSS Statistics (version 26). Given the analytical-replicate design (triplicate injections of a single pooled sample per group), formal normality and homogeneity testing was not statistically meaningful; diagnostics were used only to screen for gross outliers prior to descriptive analysis. For time-dependent changes within each group, repeated-measures analysis of variance (RM-ANOVA) was applied, with the storage time point (Days 1, 7, 14, and 21) as the within-subject factor. Sphericity was assessed using Mauchly’s test, and the Greenhouse–Geisser correction was applied where the sphericity assumption was violated. Post hoc pairwise comparisons were performed using the Bonferroni adjustment, and results are reported using letter-based grouping (different letters indicate significant differences, *p* < 0.05). Effect size is reported as partial eta-squared (partial *η*^2^); because replication was at the analytical level, *η*^2^ values are reported descriptively to indicate the magnitude of the within-sample temporal trend and are not interpreted against conventional biological effect-size thresholds. A nominal threshold of *p* < 0.05 was used only to describe patterns within the analytical-replicate dataset; however, these outputs were not interpreted as population-level statistical inference because biological replication was not resolved after pooling. Because replication was performed at the analytical level, the repeated-measures ANOVA, partial *η*^2^, and post hoc comparisons describe the consistency and magnitude of the measured within-sample temporal trend; they are not intended as tests of biological reproducibility or as a basis for population-level inference.

To complement statistical interpretation, an “apparent reduction (%)” was calculated as the relative decrease in the operationally recoverable MP-associated residue fraction between Day 1 and Day 21. This metric is explicitly relative and describes the change in the analytically recoverable MP fraction over time; it does not represent a validated absolute mass measurement, polymer degradation, or mineralization. Due to the limited number of replicates, this parameter was interpreted cautiously and used primarily to support trend-based comparisons among treatment groups.

For Py-GC/MS interpretation, polymer-specific assessment was based primarily on blank-aware EIC evaluation, since TIC signals may include matrix- and procedure-derived contributions. As stated above, EIC signal intensities are not compared across time points; all chromatographic results are interpreted as presence/absence evidence for the target polymers.

## 3. Results

### 3.1. Viability of Bifidobacterium infantis During Storage

**Figure 1:**
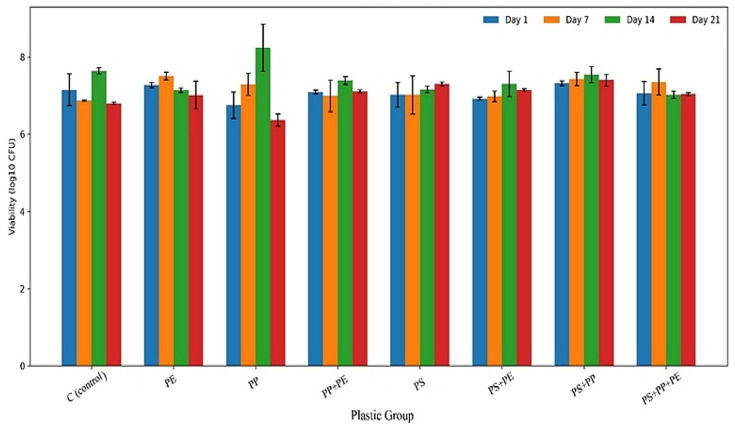
Grouped bar chart of *B. longum* subsp. *infantis* viability (mean ± *SE*, log_10_ CFU) across storage days by plastic group. Bars represent mean values ± standard error of three analytical replicate measurements (*n* = 3). No consistent time-dependent decline in viability was observed across most groups, indicating sustained probiotic survival under the experimental conditions. Abbreviations: PE (polyethylene), PP (polypropylene), PS (polystyrene).

The viability of *B. longum* subsp. *infantis* in yogurt samples containing different MP groups is presented in Table 1 and Figure 1. Across all experimental groups, viable counts remained within the range of approximately 6.3–8.2 log_10_ CFU/g throughout the 21-day refrigerated storage period.

Repeated-measures ANOVA indicated that storage time did not significantly affect viability in most groups (*p* > 0.05), including PE, PS, and mixed-polymer combinations (Table 2). A statistically significant time effect was observed only in the PP group (*p* = 0.0366), with a large effect size (partial *η*^2^ = 0.735), indicating greater variability across storage days in this group. PS-containing groups showed comparatively low temporal variability (partial *η*^2^ = 0.072–0.266) (Table 2).

### 3.2. Storage-Dependent Changes in Operationally Recoverable MP-Associated Residues

**Figure 2:**
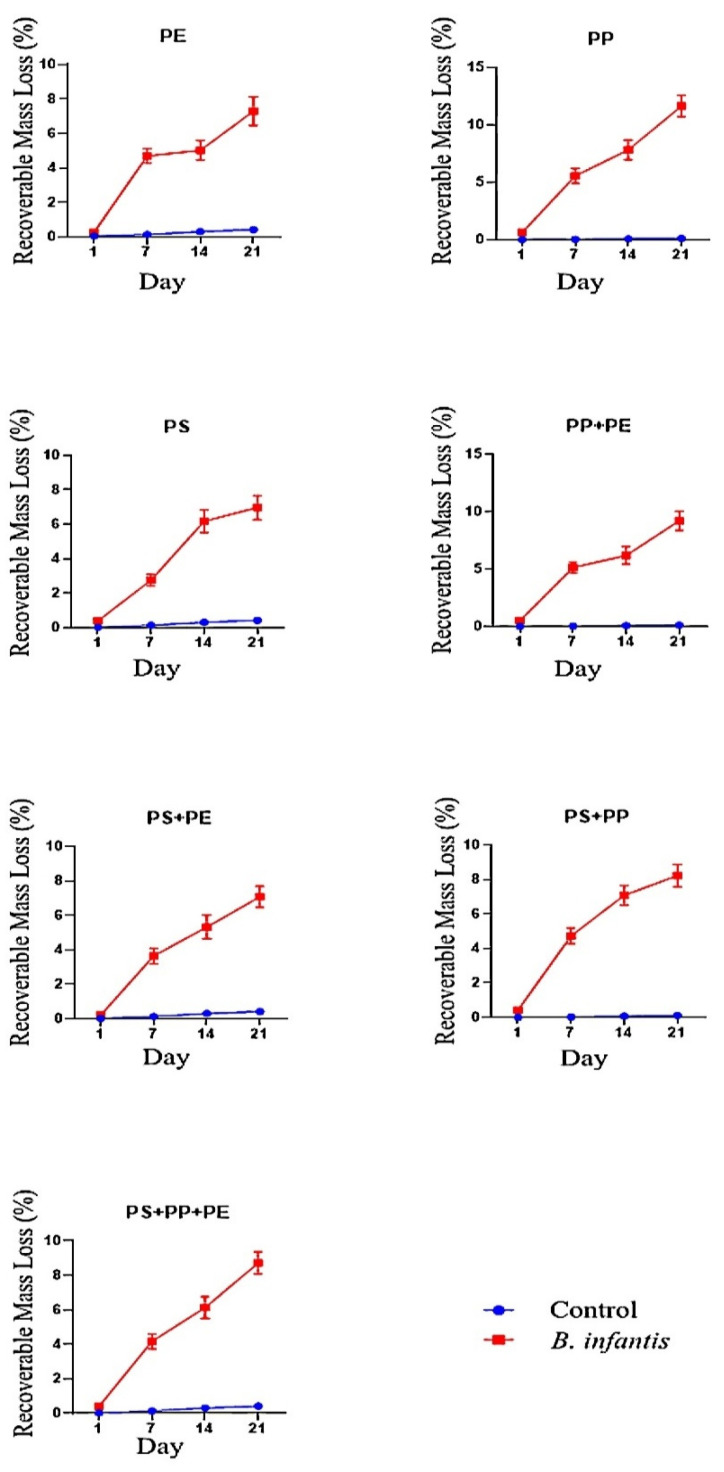
Relative decrease (%) in operationally recoverable MP-associated residues during refrigerated storage in yogurt samples containing microplastics and *B. longum* subsp. *infantis*. Percentage changes were calculated relative to Day 1 values. Trends represent reductions in operationally recoverable residues under the applied extraction conditions and should not be interpreted as validated absolute polymer loss, degradation, mineralization, or biological removal.

Changes in operationally recoverable MP-associated residue fractions during storage are shown in Table 3 and Table 4, Figure 2. In control groups, polymer mass remained stable throughout the storage period (*p* > 0.05), with minimal variation and low effect sizes (partial *η*^2^ ≤ 0.411).

This metric reflects changes in operationally recoverable MP-associated residue fractions and does not indicate polymer degradation, mineralization, or biological removal. The apparent reduction (%) values indicate a polymer-dependent trend in the decrease in operationally recoverable MP-associated residues during storage. The highest reduction was observed in PP-containing samples, whereas PS exhibited the lowest reduction. Mixed-polymer systems showed intermediate values, suggesting partial overlap in the recovery-change behavior across polymer types.

In contrast, all groups containing *B. longum* subsp. *infantis* exhibited consistent, time-dependent reductions in operationally recoverable MP-associated residue fractions across the storage period, with the corresponding within-sample trend statistics summarized in Table 3 (partial *η*^2^ = 0.819–0.953). Among single-polymer systems, the largest reduction was observed in PP (49.7 ± 0.1 mg to 44.2 ± 0.9 mg), followed by PE (49.9 ± 0.1 mg to 46.3 ± 0.6 mg) and PS (49.6 ± 0.1 mg to 46.5 ± 1.0 mg).

Comparable decreasing trends were observed in mixed-polymer groups, including PS + PP (partial *η*^2^ = 0.917), PS + PP + PE (0.892), PP + PE (0.881), and PS + PE (0.819), indicating consistent reductions across different polymer combinations.

### 3.3. Py-GC/MS Qualitative Confirmation of Polymer Identity

**Figure 3:**
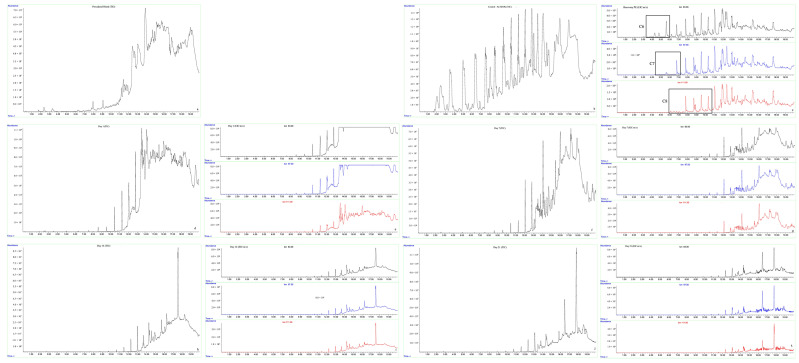
Total ion chromatograms (TICs) and polyethylene (PE)-targeted extracted-ion chromatograms (EICs) of yogurt samples across refrigerated storage. Panels (**a**–**k**) represent the different conditions and time points. Panel (**a**) corresponds to the procedural blank and (**b**) to the polymer-free control (both TICs); panel (**c**) shows the PE recovery control (EIC). Panels (**d**,**f**,**h**,**j**) show TIC profiles for Days 1, 7, 14, and 21, respectively, while panels (**e**,**g**,**i**,**k**) show the corresponding EIC profiles (*m*/*z* 83, 97, 111). In the recovery-control panel (**c**), the PE-diagnostic homologous ion series (labelled C6–C8) is indicated to mark the characteristic PE fragmentation pattern. These diagnostic ions remained detectable at all sampled time points, confirming retention of polymer identity throughout storage. Because no internal standard was used, EIC signal intensities are not compared across panels, and no inference is drawn from differences in peak height or scale. In the Day 1 EIC panel (**e**), the *m*/*z* 83 and 97 traces exceed the linear detector range (off-scale plateau beyond ~13.5 min) and are not interpreted; this saturation further illustrates why EIC intensities are not compared across panels or time points. Interpretations are based primarily on EIC data, as TIC signals may include matrix-related contributions.

**Figure 4:**
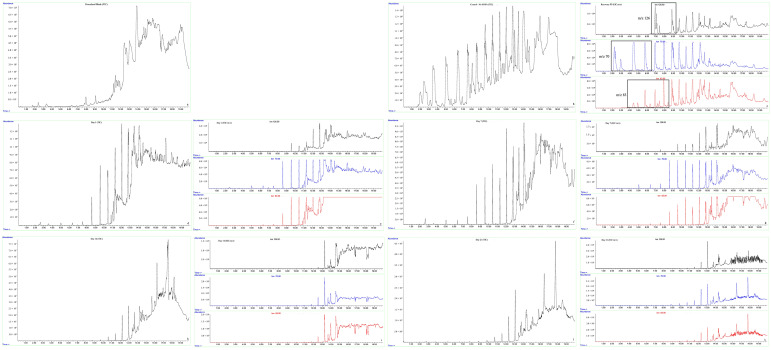
Total ion chromatograms (TICs) and polypropylene (PP)-targeted extracted-ion chromatograms (EICs) of yogurt samples across refrigerated storage. Panels (**a**–**k**) represent the different conditions and time points. Panel (**a**) corresponds to the procedural blank and (**b**) to the polymer-free control (both TICs); panel (**c**) shows the PP recovery control (EIC). Panels (**d**,**f**,**h**,**j**) show TIC profiles for Days 1, 7, 14, and 21, respectively, while panels (**e**,**g**,**i**,**k**) show the corresponding EIC profiles (*m*/*z* 70, 83, 126). In the recovery-control panel (**c**), the PP-diagnostic ion regions (*m*/*z* 70, 83, 126) are indicated to mark the characteristic PP fragmentation pattern. These diagnostic ions remained detectable at all sampled time points, confirming retention of polymer identity throughout storage. Because no internal standard was used, EIC signal intensities are not compared across panels, and no inference is drawn from differences in peak height or scale; in the Day 1 EIC panel (**e**), the *m*/*z* 83 trace exceeds the linear detector range beyond ~13 min and is not interpreted. Interpretations are based primarily on EIC data, as TIC signals may include matrix-related contributions.

**Figure 5:**
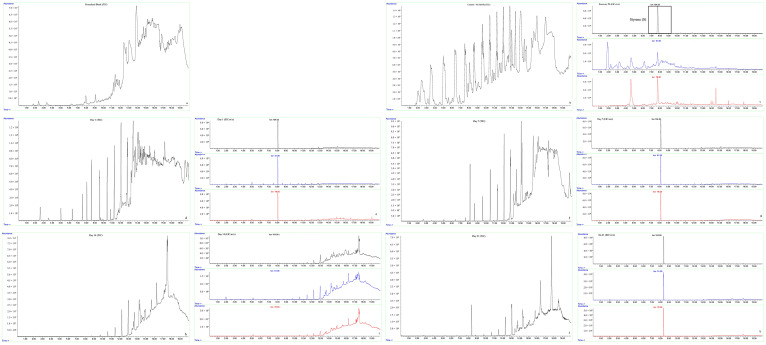
Total ion chromatograms (TICs) and polystyrene (PS)-targeted extracted-ion chromatograms (EICs) of yogurt samples across refrigerated storage. Panels (**a**–**k**) represent the different conditions and time points. Panel (**a**) corresponds to the procedural blank and (**b**) to the polymer-free control (both TICs); panel (**c**) shows the PS recovery control (EIC). Panels (**d**,**f**,**h**,**j**) show TIC profiles for Days 1, 7, 14, and 21, respectively, while panels (**e**,**g**,**i**,**k**) show the corresponding EIC profiles (*m*/*z* 51, 78, 104). In the recovery-control panel (**c**), the characteristic styrene monomer peak (*m*/*z* 104, with fragment ions *m*/z 51 and 78) is indicated as the diagnostic marker for PS. This diagnostic signal remained detectable at all sampled time points, confirming retention of polymer identity throughout storage. Because no internal standard was used, EIC signal intensities are not compared across panels, and no inference is drawn from differences in peak height or scale. Interpretations are based primarily on EIC data, as TIC signals may include matrix-related contributions.

Py-GC/MS analysis was used solely for qualitative confirmation of polymer identity in yogurt samples at each time point. Each analytical run included a procedural blank, a polymer-free matrix control, and a spiked recovery control for each polymer type; these controls confirmed that polymer-specific EIC signals remained detectable after the full extraction protocol and that diagnostic ions in experimental samples were attributable to added polymers rather than to procedural artefacts or matrix background (panels a–c, a–c, and a–c in Figure 3, Figure 4 and Figure 5, respectively). EIC signal intensities are not compared across time points, and no semi-quantitative inference about polymer mass changes is drawn from the chromatographic data.

Polymer-targeted extracted ion chromatograms (EICs) confirmed the presence of polymer-specific signals at all four time points (Days 1, 7, 14, and 21). Characteristic diagnostic ions for PE (*m*/*z* 83, 97, 111), PP (*m*/*z* 70, 83, 126), and PS (*m*/*z* 51, 78, 104) were identifiable in all MP-containing samples, confirming polymer identity throughout the storage period (Figure 3, Figure 4 and Figure 5).

No semi-quantitative comparison of EIC signal intensities across time points is made; all chromatographic interpretation is limited to qualitative polymer identification based on diagnostic ion presence and fragmentation pattern recognition.

#### 3.3.1. Qualitative Confirmation of PE-Specific Signals

PE-targeted EICs (*m*/*z* 83, 97, 111) confirmed the presence of polyethylene-specific fragmentation ions at all four time points (Figure 3). Characteristic PE signals were identifiable across the storage period, supporting polymer identity. An increase in background chromatographic noise was qualitatively noted at later time points; however, no quantitative inference is drawn from these observations.

#### 3.3.2. Qualitative Confirmation of PP-Specific Signals

PP-targeted EICs (*m*/*z* 70, 83, 126) confirmed the presence of polypropylene-specific fragmentation ions at all sampling time points (Figure 4). Characteristic PP signals were consistently identifiable throughout the 21-day storage period, confirming polymer identity in PP-containing groups.

#### 3.3.3. Qualitative Confirmation of PS-Specific Signals

PS-targeted EICs (*m*/*z* 51, 78, 104) showed the highest specificity among the three polymers, with distinct polymer-specific fragmentation patterns observed in PS-containing samples and limited interference from background signals (Figure 5). The styrene monomer ion (*m*/*z* 104) was consistently present at all time points, providing reliable qualitative confirmation of polystyrene identity throughout the storage period.

#### 3.3.4. Integrated Interpretation of Operational Gravimetry and Qualitative Py-GC/MS Data

Across all polymer types, the operationally recoverable MP-associated residue fraction showed consistent time-dependent reductions (Section 3.2), while Py-GC/MS confirmed polymer identity at all sampling time points. The persistent detectability of polymer-specific EIC signals across the 21-day storage period indicates that polymer-specific diagnostic ions remained detectable throughout storage; the qualitative nature of the data does not, however, allow conclusions about polymer persistence, degradation, or biological removal to be drawn.

These results indicate that reductions in operationally recoverable MP-associated residue fractions coexisted with persistent polymer identity as confirmed by Py-GC/MS. Because signal intensities are not compared semi-quantitatively in the absence of an internal standard, Py-GC/MS data support polymer presence but cannot independently characterize the nature or extent of any change.

## 4. Discussion

Building on earlier exploratory work suggesting that *Bifidobacterium* may interact with microplastics [25], the present study provides a systematic, food-matrix-based evaluation of time-dependent changes in the recoverable MP fraction within a real yogurt system in the presence of the probiotic *B. longum* subsp. *infantis*, combining recoverable-fraction monitoring with polymer-specific Py-GC/MS identity confirmation.

Throughout the 21-day refrigerated storage period, *B. longum* subsp. *infantis* remained viable within the probiotic-relevant range (6.3–8.2 log_10_ CFU/g), exceeding the minimum threshold required for functional activity in food systems [36], indicating that the yogurt matrix supported sustained metabolic activity. This observation is consistent with previous reports demonstrating the persistence of bifidobacteria in fermented dairy systems and their tolerance to storage-associated stress conditions [37,38]. Collectively, the results demonstrate that probiotic-associated processes coincide with reductions in operationally recoverable MP-associated residue fractions and concurrent changes in polymer-related analytical signals during storage.

A central observation of this study is the consistent time-dependent reduction in operationally recoverable MP-associated residue fractions across all MP-containing groups in the presence of *Bifidobacterium longum* subsp. *infantis*, whereas recoverable fractions in the corresponding control systems remained essentially stable over the same period. This supports an association between probiotic activity and the observed changes, rather than passive storage effects.

The temporal pattern of change supports a non-uniform interaction process. Reductions were most pronounced during Days 1–7 and slowed toward Day 21. These non-linear kinetics—rapid initial decline followed by stabilization—are more consistent with a saturable surface-binding process than with continuous degradation, which would be expected to progress steadily over time [23,24].

Several non-degradative mechanisms must be acknowledged as plausible contributors. First, adsorption onto bacterial cell surfaces or extracellular polymeric substances (EPS) offers a parsimonious explanation. Zhao et al. [24] showed that lactic acid bacteria bind nanoplastics through hydrophobic, electrostatic, hydrogen-bonding, and van der Waals interactions. Resulting bacteria–particle aggregates would sediment during cold storage, reducing the recoverable fraction without chemical modification of the polymer backbone.

Second, milk protein interactions provide a further non-degradative route. Brouwer et al. [39] demonstrated that MPs acquire a protein corona under digestive conditions. In yogurt, casein micelles undergoing acidification-driven structural change may similarly aggregate with MP particles, shifting particle size distribution and filterability independently of any polymer change [39,40,41]. Chitosan has recently been shown to physically entrap MPs under in vitro gastric conditions [42], further supporting matrix-capture rather than degradation as a contributor to reduced recoverable fractions.

Third, progressive pH decrease during storage may partially inhibit Proteinase K, which is optimally active at pH 7.5–8.0. The oxidative treatment steps (H_2_O_2_/H_2_SO_4_) are also pH-sensitive [43]. Such pH-dependent shifts in extraction efficiency could produce time-dependent yield reductions independently of any polymer change. Spiked recovery controls confirmed qualitative polymer detectability after extraction; however, pH-dependent recovery variation was not directly tested.

Finally, matrix-derived residues—lipids, proteins, and mineral salts surviving digestion—may contribute to the gravimetric filter residue in storage-time-dependent ways. The polymer-free matrix control provides a reference for these non-polymer contributions at each time point [6,33].

Taken together, surface adsorption, protein-corona aggregation, and pH-dependent extraction effects argue against bulk polymer degradation as the primary driver of the reductions. All three are consistent with the saturable kinetics observed. The interpretation remains inferential, as the experimental design cannot directly distinguish among these contributors. Changes are therefore described conservatively as reductions in the recoverable residue fraction under the applied protocol, and causal mechanistic conclusions are avoided.

Reduction magnitude differed across polymer types: PP showed the greatest apparent decrease (≈11.1%), followed by PE (≈7.1%) and PS (≈6.2%) (Figure 2). This polymer-dependent pattern is consistent with reports that polymer structure influences particle interactions with surrounding matrix and microbial components [44,45]. PP’s branched structure may offer greater accessibility for surface interactions. The aromatic backbone of PS, by contrast, confers relative resistance to chemical modification [30]. The findings underscore the relevance of multi-polymer contamination scenarios in food safety assessments.

Py-GC/MS served to confirm polymer identity rather than to quantify it. Procedural blanks, polymer-free matrix controls, and spiked recovery controls confirmed that diagnostic ions in experimental samples were attributable to added polymers, not to contamination or matrix background. Polymer-specific signals remained detectable at all sampling points, supporting the continued presence of the polymers throughout storage. However, because the data are qualitative, neither polymer persistence nor partial degradation can be formally established or excluded. This interpretive limitation is consistent with the broader literature: microplastic changes typically involve surface oxidation, limited chain scission, and fragmentation rather than complete mineralization [29,31,46], and microbial interactions with polymer surfaces can produce partial surface modification without proportional bulk mass loss [45,47,48]. Accordingly, the chromatographic data confirm polymer identity at each time point but cannot distinguish an actual change in polymer mass from variation in extraction efficiency or matrix interference.

From an applied perspective, these findings highlight yogurt as a relevant food model in which probiotic functionality may extend beyond conventional health benefits to include modulation of emerging contaminants. This possibility is consistent with a growing body of work on probiotic–contaminant interactions. Probiotic strains have long been shown to bind and biotransform diverse organic and inorganic contaminants—including mycotoxins, polycyclic aromatic hydrocarbons, and heavy metals—through cell-wall adsorption and detoxification mechanisms [17,18]. Extending this concept to plastic particles, Bazeli et al. [12] reviewed whether probiotics could mitigate polystyrene micro- and nanoplastic toxicity, concluding that binding and surface-association represent plausible protective routes. The reductions observed here are consistent with that framework. Rather than degrading the polymers, *B. longum* subsp. *infantis* may bind or sequester MP particles at the cell surface, paralleling contaminant-binding behavior documented for other probiotic strains [17,18,24]. This positions the present observation within an established line of probiotic-detoxification research while underscoring that the protective relevance of such binding for human exposure remains to be demonstrated. The broader role of gut bacteria in mitigating microplastic effects within the digestive system has been reviewed recently [49], situating the present findings within this expanding field.

MPs have been widely documented across dairy products and throughout the production chain [50,51], with PP, PE, and PS among the most frequently detected types [6,7,8]. Even moderate storage-associated reductions in recoverable fractions could contribute to lowering dietary exposure, particularly in high-consumption settings such as Türkiye [52]. Dairy production systems also represent a critical node in the environmental–animal–human interface. Livestock are exposed to MPs through contaminated feed, silage wrapping, and drinking water, and MPs have been documented in bovine milk, blood, and edible tissues, establishing a farm-to-fork transfer pathway [9,10,11]. These livestock and farm-to-fork considerations provide important background. However, they extend beyond the scope of this in vitro study; the observed changes pertain specifically to the finished yogurt matrix under refrigerated storage.

The present study examines probiotic–microplastic interactions within a real fermented dairy system under realistic storage conditions. This contrasts with the earlier observation by Bozkurt et al. [25], which was exploratory and obtained under conditions inconsistent with the organism’s anaerobic physiology. The present findings should therefore be regarded as exploratory and hypothesis-generating rather than confirmatory.

MP–microbiota interactions also extend beyond the food matrix to animal gut health. In ruminants, MPs have been shown to disrupt the rumen microbial ecosystem, impairing fermentation activity, reducing fiber digestibility, and altering the Firmicutes: Bacteroidetes ratio [14,15]. In poultry, MP exposure has been linked to reduced gut microbiota diversity and impaired intestinal barrier integrity [16]. These findings suggest that gastrointestinal microbiota in food-producing animals may both be affected by and influence the behavior of ingested MPs—a bidirectional relationship with implications for animal welfare and product safety [53,54]. These in vivo livestock processes provide broader context but are not addressed by the present study. The in vitro data reported here cannot be extrapolated to conclusions regarding animal or environmental health; such links require future validation using in vivo models or simulated gut-culture systems.

As a broader interpretive framework, the findings may be contextualized within a One Health perspective, which recognizes the inseparability of environmental, animal, and human health [26,27]. MPs enter agricultural systems through environmental contamination, accumulate in livestock through feed and water exposure, and ultimately reach human consumers via animal-derived food products [9,10,53]. Probiotic microorganisms—present across this continuum, from animal gut flora to fermented dairy products—represent an understudied biological interface within this chain. Understanding the capacity of specific strains to modulate MP fractions in food matrices may inform future risk mitigation strategies bridging food microbiology, veterinary science, and environmental health.

Several limitations should be acknowledged. Regarding replication, three independent production batches per group were pooled into a single composite sample, which was then analyzed in triplicate. Replication was therefore analytical rather than biological. Reported standard errors reflect instrumental repeatability and should not be interpreted as evidence of low biological variability. RM-ANOVA, *p*-values, and partial *η*^2^ describe within-sample temporal consistency, not biological reproducibility or population-level effects. Confirmatory studies with independently analyzed replicates (n ≥ 6 per group) and non-parametric or bootstrap approaches are required to establish statistical and biological robustness.

Mechanistic resolution is constrained by several analytical absences. GPC/SEC was not performed, precluding direct confirmation of polymer chain scission. SEM and FTIR analyses were not conducted, limiting surface morphological and functional group characterization. Particle size distribution of recovered particles was not determined. No internal standard was employed in Py-GC/MS; signal intensities are therefore not compared across time points, and all chromatographic data are interpreted as qualitative polymer identity confirmation only.

Regarding extraction validation, procedural blanks, polymer-free matrix controls, and spiked recovery controls confirmed qualitative polymer detectability after the full extraction protocol. However, formal quantitative recovery efficiency with matrix-matched standards, absolute mass balance verification, and pH- or time-point-specific recovery experiments were not performed. Non-polymer matrix residue contributions to the gravimetric filter residue were controlled only indirectly through the polymer-free control group. Consequently, gravimetric values are relative, trend-level indicators—not validated absolute polymer masses—and the small magnitude of observed changes (~6–11%) warrants corresponding caution.

A final limitation concerns matrix evolution during storage. Yogurt is a dynamic protein–lipid–mineral gel system. Storage-dependent changes in acidity, casein aggregation, microbial metabolites, and filtration behavior may alter extraction efficiency and residue formation independently of any polymer change. The operational gravimetric decreases reported here should therefore be interpreted as changes in recoverability of MP-associated residues, not as evidence of polymer degradation or removal.

Future studies should incorporate orthogonal analytical approaches—including FTIR, GPC/SEC, and electron microscopy—alongside quantitative Py-GC/MS standardization with internal standards. Expanding investigations to additional probiotic strains and food matrices will be important for assessing the broader relevance of these observations. Such efforts are essential for advancing mechanistic understanding of probiotic–microplastic interactions in complex food systems.

## 5. Conclusions

This exploratory in vitro study showed that yogurt samples containing *B. longum* subsp. *infantis* exhibited storage-dependent decreases in operationally defined, digestion-resistant recoverable residues associated with PP, PE, and PS microplastics during 21 days of refrigerated storage. Among the tested formulations, PP-containing samples showed the greatest apparent decrease, whereas PS-containing samples showed the smallest. *B. longum* subsp. *infantis* remained viable throughout storage, indicating that the yogurt matrix supported probiotic survival under the tested conditions.

Py-GC/MS confirmed the qualitative detectability of polymer-specific signals at all sampled time points. Because no internal standard, matrix-matched recovery validation, pH-dependent recovery assessment, or biological replication at the extraction level was included, the observed gravimetric trends should not be interpreted as validated polymer mass loss, degradation, mineralization, or biological removal. Instead, the results support a cautious interpretation involving altered extractability, filterability, adsorption, aggregation, or matrix-associated recovery shifts.

Overall, the findings should be regarded as preliminary and hypothesis-generating. They are relevant to the behavior of microplastic-associated residues within the tested yogurt matrix but cannot be extrapolated to livestock exposure, human dietary risk, or farm-to-fork transfer without additional validated quantitative methods, independent biological replication, particle-size characterization, pH/time-dependent recovery experiments, and more realistic food-chain or gastrointestinal models.

## Figures and Tables

**Table 1:** Viability of *B. longum* subsp. *infantis* in yogurt samples containing different microplastic formulations during refrigerated storage (log_10_ CFU/g, Mean ± SE).

Plastic Group	n	1. Day (log_10_ Mean ± *SE*)	7. Day (log_10_ Mean ± *SE*)	14. Day (log_10_ Mean ± *SE*)	21. Day (log_10_ Mean ± *SE*)	*p* (Time)	Partial *η*^2^
K (control)	3	7.15 ± 0.41	6.87 ± 0.02	7.64 ± 0.08	6.80 ± 0.03	0.0744	0.660
PE	3	7.27 ± 0.06 ^a^	7.51 ± 0.10 ^a^	7.14 ± 0.06 ^a^	7.01 ± 0.36 ^a^	0.3338	0.410
PP	3	6.75 ± 0.34 ^ab^	7.29 ± 0.29 ^ab^	8.24 ± 0.61 ^a^	6.37 ± 0.16 ^b^	0.0366	0.735
PP + PE	3	7.09 ± 0.05 ^a^	6.99 ± 0.41 ^a^	7.39 ± 0.10 ^a^	7.11 ± 0.04 ^a^	0.6652	0.216
PS	3	7.02 ± 0.32 ^a^	7.02 ± 0.49 ^a^	7.16 ± 0.09 ^a^	7.30 ± 0.05 ^a^	0.9231	0.072
PS + PE	3	6.92 ± 0.04 ^a^	6.98 ± 0.14 ^a^	7.30 ± 0.33 ^a^	7.15 ± 0.03 ^a^	0.5727	0.266
PS + PP	3	7.32 ± 0.06 ^a^	7.43 ± 0.17 ^a^	7.54 ± 0.21 ^a^	7.40 ± 0.15 ^a^	0.8332	0.126
PS + PP + PE	3	7.06 ± 0.30 ^a^	7.35 ± 0.34 ^a^	7.02 ± 0.09 ^a^	7.04 ± 0.04 ^a^	0.6847	0.206

Values are expressed as log_10_ CFU/g (mean ± standard error of three analytical replicate measurements, *n* = 3). Different superscript letters within the same row indicate differences observed among analytical replicate measurements within the descriptive repeated-measures framework. Because the values derive from analytical replicates of pooled composite samples, these groupings should not be interpreted as evidence of biological reproducibility or population-level statistical significance. The column “*p* (time)” represents the significance of the storage-time effect, and partial eta-squared (partial *η*^2^) indicates the corresponding effect size. Abbreviations: PE (polyethylene), PP (polypropylene), PS (polystyrene). The *p*-values and partial *η*^2^ values are reported only as descriptive indicators of temporal consistency within analytical replicate measurements and are not interpreted as evidence of biological reproducibility or population-level effects.

**Table 2:** Results of repeated-measures ANOVA evaluating the effect of storage time on *B. longum* subsp. *infantis* viability within each microplastic group.

Plastic Group	F	df1	df2	*p*	Partial *η*^2^
K (control)	3.879	3	6	0.0744	0.660
PE	1.390	3	6	0.3338	0.410
PP	5.553	3	6	0.0366	0.735
PP + PE	0.550	3	6	0.6652	0.216
PS	0.156	3	6	0.9231	0.072
PS + PE	0.724	3	6	0.5727	0.266
PS + PP	0.287	3	6	0.8332	0.126
PS + PP + PE	0.519	3	6	0.6847	0.206

Analyses were performed separately for each plastic formulation using log_10_-transformed viability data across storage days (1, 7, 14, and 21). F, degrees of freedom (df1, df2), and *p*-values are reported for the within-subject factor (storage time). Partial eta-squared (partial *η*^2^) represents the magnitude of the time effect. Abbreviations: PE (polyethylene), PP (polypropylene), PS (polystyrene). The *p*-values and partial *η*^2^ values are reported only as descriptive indicators of temporal consistency within analytical replicate measurements and are not interpreted as evidence of biological reproducibility or population-level effects.

**Table 3:** Storage-dependent changes in operationally recoverable MP-associated residue fractions in yogurt samples containing single and combined microplastic formulations in the presence and absence of *B. longum* subsp. *infantis*. Values are normalized to a 100 g yogurt-equivalent basis and should not be interpreted as validated absolute polymer masses.

MPs	Group	Day 1 (mg/100 g Yogurt-Equivalent)	Day 7 (mg/100 g Yogurt-Equivalent)	Day 14 (mg/100 g Yogurt-Equivalent)	Day 21 (mg/100 g Yogurt-Equivalent)	*p* (Time)	Partial *η*^2^
PE	MP-only control (no *B. longum* subsp. *infantis*)	50.0 ± 0.1	49.9 ± 0.1	49.8 ± 0.2	49.7 ± 0.2	0.3318	0.411
	*B. longum* subsp. *infantis*	49.9 ± 0.1 ^a^	47.7 ± 0.1 ^b^	47.5 ± 0.1 ^b^	46.3 ± 0.6 ^c^	0.0003	0.949
PP	MP-only control (no *B. longum* subsp. *infantis*)	50.0 ± 0.1	50.0 ± 0.1	49.9 ± 0.2	49.9 ± 0.2	0.9749	0.033
	*B. longum* subsp. *infantis*	49.7 ± 0.1 ^a^	47.2 ± 0.2 ^b^	46.1 ± 0.2 ^c^	44.2 ± 0.9 ^d^	0.0002	0.953
PS	MP-only control (no *B. longum* subsp. *infantis*)	50.0 ± 0.1	49.9 ± 0.1	49.8 ± 0.1	49.7 ± 0.3	0.6346	0.233
	*B. longum* subsp. *infantis*	49.6 ± 0.1 ^a^	48.6 ± 0.2 ^b^	46.9 ± 0.3 ^c^	46.5 ± 1.0 ^c^	0.0075	0.845
PP + PE	MP-only control (no *B. longum* subsp. *infantis*)	50.0 ± 0.1	50.0 ± 0.1	49.9 ± 0.2	49.9 ± 0.2	0.9682	0.039
	*B. longum* subsp. *infantis*	49.6 ± 0.1 ^a^	47.4 ± 0.2 ^b^	46.9 ± 0.2 ^c^	45.4 ± 1.0 ^cd^	0.0035	0.881
PS + PE	MP-only control (no *B. longum* subsp. *infantis*)	50.0 ± 0.1	49.9 ± 0.1	49.8 ± 0.1	49.7 ± 0.2	0.6816	0.207
	*B. longum* subsp. *infantis*	49.7 ± 0.1 ^a^	48.1 ± 0.3 ^b^	47.3 ± 0.2 ^c^	46.4 ± 1.1 ^c^	0.0119	0.819
PS + PP	MP-only control (no *B. longum* subsp. *infantis*)	50.0 ± 0.1	50.0 ± 0.1	49.9 ± 0.2	49.9 ± 0.2	0.9592	0.046
	*B. longum* subsp. *infantis*	49.6 ± 0.1 ^a^	47.6 ± 0.2 ^b^	46.4 ± 0.1 ^c^	45.8 ± 0.8 c^d^	0.0012	0.917
PS + PP + PE	MP-only control (no *B. longum* subsp. *infantis*)	50.0 ± 0.1	49.9 ± 0.1	49.8 ± 0.2	49.7 ± 0.3	0.5503	0.279
	*B. longum* subsp. *infantis*	49.5 ± 0.1 ^a^	47.9 ± 0.2 ^b^	46.9 ± 0.2 ^c^	45.6 ± 0.8 ^cd^	0.0027	0.892

Values are presented as mean ± standard error of three analytical (technical) replicate measurements (*n* = 3). “Control” groups refer to yogurt samples containing the corresponding microplastics without *B. longum* subsp. *infantis*, whereas “*B. longum* subsp. *infantis*” groups include probiotic supplementation. Different superscript letters within each *B. longum* subsp. *infantis* row indicate differences observed among analytical replicate measurements under the descriptive repeated-measures framework. Because the dataset is based on triplicate injections of pooled composite samples, these groupings should be interpreted as descriptive within-sample temporal patterns rather than as evidence of biological reproducibility or population-level effects. The “*p* (time)” column represents the statistical significance of the storage-time effect, and partial eta-squared (partial *η*^2^) is reported descriptively to indicate the magnitude of the within-sample temporal trend; given the analytical-replicate design, these values are not interpreted as biological effect sizes. Changes are reported as variations in operationally recoverable MP-associated residue fraction under the applied extraction protocol, and should not be interpreted as direct evidence of complete polymer degradation. Abbreviations: PE (polyethylene), PP (polypropylene), PS (polystyrene). The *p*-values and partial *η*^2^ values are reported only as descriptive indicators of temporal consistency within analytical replicate measurements and are not interpreted as evidence of biological reproducibility or population-level effects.

**Table 4:** Apparent reduction (%) in operationally recoverable MP-associated residues and descriptive comparison among groups. The values are trend-level, normalized, operational indicators and do not demonstrate polymer degradation, mineralization, or biological removal.

Group	Polymer	Day 1 (mg/100 g Yogurt-Equivalent)	Day 21 (mg/100 g Yogurt-Equivalent)	Apparent Reduction (%)	Grouping
PP + *B. longum* subsp. *infantis*	PP	49.7 ± 0.1	44.2 ± 0.9	11.1	^a^
PP + PE + *B. longum* subsp. *infantis*	PP + PE	49.6 ± 0.1	45.4 ± 1.0	8.5	^ab^
PS + PP + PE + *B. longum* subsp. *infantis*	All	49.5 ± 0.1	45.6 ± 0.8	7.9	^ab^
PS + PP + *B. longum* subsp. *infantis*	PS + PP	49.6 ± 0.1	45.8 ± 0.8	7.5	^ab^
PE + *B. longum* subsp. *infantis*	PE	49.9 ± 0.1	46.3 ± 0.6	7.1	^b^
PS + PE + *B. longum* subsp. *infantis*	PS + PE	49.7 ± 0.1	46.4 ± 1.1	6.5	^b^
PS + *B. longum* subsp. *infantis*	PS	49.6 ± 0.1	46.5 ± 1.0	6.2	^b^

Apparent Reduction (%) was calculated as (Day 1 − Day 21)/Day 1 × 100. Different superscript letters (a,b) denote differences observed among groups within the descriptive analytical-replicate dataset. Because the apparent reduction values were derived from pooled composite samples measured by analytical replicate injections, these groupings should not be interpreted as confirmatory evidence of biological or population-level differences.

## Data Availability

The data presented in this study are available from the corresponding author upon reasonable request. Due to the large size and specialized format of the Py-GC/MS datasets, they are not publicly deposited but can be provided for verification and further analysis.

## Abbreviations

The following abbreviations are used in this manuscript:

MPs	Microplastics
PP	Polypropylene
PE	polyethylene
PS	polystyrene
Py-GC/MS	pyrolysis–gas chromatography/mass spectrometry
PAHs	polycyclic aromatic hydrocarbons
ATCC	American Type Culture Collection
TMAH	tetramethylammonium hydroxide
BHT	butylated hydroxytoluene
TCB	1,2,4-trichlorobenzene
TICs	Total ion chromatograms
EICs	extracted-ion chromatograms
SE	standard error

## References

[1] Geyer R., Letcher T.M. (2020). Production, Use, and Fate of Synthetic Polymers. Plastic Waste and Recycling.

[2] Rillig M.C. (2012). Microplastic in Terrestrial Ecosystems and the Soil?. Environ. Sci. Technol..

[3] Wang H.-T., Ding J., Xiong C., Zhu D., Li G., Jia X.-Y., Zhu Y.-G., Xue X.-M. (2019). Exposure to Microplastics Lowers Arsenic Accumulation and Alters Gut Bacterial Communities of Earthworm *Metaphire californica*. Environ. Pollut..

[4] Lin Q., Zhao S., Pang L., Sun C., Chen L., Li F. (2022). Potential Risk of Microplastics in Processed Foods: Preliminary Risk Assessment Concerning Polymer Types, Abundance, and Human Exposure of Microplastics. Ecotoxicol. Environ. Saf..

[5] Garrido Gamarro E., Costanzo V. (2022). Microplastics in Food Commodities: A Food Safety Review on Human Exposure Through Dietary Sources.

[6] Ling X., Cheng J., Yao W., Qian H., Ding D., Yu Z., Xie Y., Yang F. (2024). Identification and Visualization of Polystyrene Microplastics/Nanoplastics in Flavored Yogurt by Raman Imaging. Toxics.

[7] Zipak S., Muratoğlu K., Büyükünal S. (2022). Evaluation of Microplastic Presence in Yogurt Production Process. Kafkas Univ. Vet. Fak. Derg..

[8] Basaran B., Özçifçi Z., Akcay H.T., Aytan Ü. (2023). Microplastics in Branded Milk: Dietary Exposure and Risk Assessment. J. Food Compos. Anal..

[9] Aardema H., Vethaak A.D., Kamstra J.H., Legler J. (2024). Farm Animals as a Critical Link Between Environmental and Human Health Impacts of Micro-and Nanoplastics. Micropl. Nanopl..

[10] Urli S., Corte Pause F., Crociati M., Baufeld A., Monaci M., Stradaioli G. (2023). Impact of Microplastics and Nanoplastics on Livestock Health: An Emerging Risk for Reproductive Efficiency. Animals.

[11] Khan A., Qadeer A., Wajid A., Ullah Q., Rahman S.U., Ullah K., Safi S.Z., Ticha L., Skalickova S., Chilala P. (2024). Microplastics in Animal Nutrition: Occurrence, Spread, and Hazard in Animals. J. Agric. Food Res..

[12] Bazeli J., Banikazemi Z., Hamblin M.R., Sharafati Chaleshtori R. (2023). Could Probiotics Protect Against Human Toxicity Caused by Polystyrene Nanoplastics and Microplastics?. Front. Nutr..

[13] Meng X., Zhang J., Wang W., Gonzalez-Gil G., Vrouwenvelder J.S., Li Z. (2022). Effects of Nano-and Microplastics on Kidney: Physicochemical Properties, Bioaccumulation, Oxidative Stress and Immunoreaction. Chemosphere.

[14] Eichinger J., Seifert J., Sáenz J.S., Amin N., Lorenz S., Eckel F., Zollfrank C., Windisch W., Brugger D. (2025). The Interaction of Microplastics with the Ruminal Ecosystem in Vitro. J. Hazard. Mater..

[15] Tassone S., Kaihara H., Barbera S., Glorio Patrucco S., Issaoui R., Abid K. (2025). Low-Density Polyethylene Microplastics in the Rumen: Implications for Rumen Fermentation Dynamics and Utilization of Concentrate Feed. Animals.

[16] Zou W., Lu S., Wang J., Xu Y., Shahid M.A., Saleem M.U., Mehmood K., Li K. (2023). Environmental Microplastic Exposure Changes Gut Microbiota in Chickens. Animals.

[17] Pop O.L., Suharoschi R., Gabbianelli R. (2022). Biodetoxification and Protective Properties of Probiotics. Microorganisms.

[18] Yousefi M., Khorshidian N., Hosseini H. (2022). The Ability of Probiotic Lactobacillus Strains in Removal of Benzo[a]Pyrene: A Response Surface Methodology Study. Probiotics Antimicro. Prot..

[19] Karamese M., Aydin H., Gelen V., Sengul E., Karamese S.A. (2020). The Anti-Inflammatory, Anti-Oxidant and Protective Effects of a Probiotic Mixture on Organ Toxicity in a Rat Model. Future Microbiol..

[20] Mattarelli P., Bonaparte C., Pot B., Biavati B. (2008). Proposal to Reclassify the Three Biotypes of *Bifidobacterium longum* as Three Subspecies: *Bifidobacterium longum* subsp. *longum* subsp. Nov., *Bifidobacterium longum* subsp. *infantis* Comb. Nov. and *Bifidobacterium longum subsp*. suis comb. Nov. Int. J. Syst. Evol. Microbiol..

[21] Stewart C.J. (2021). Breastfeeding Promotes Bifidobacterial Immunomodulatory Metabolites. Nat. Microbiol..

[22] Huang J., Cheng H. (2025). Effects of *Bifidobacterium* on Metabolic Parameters in Overweight or Obesity Adults: A Systematic Review and Meta-Analysis. Front. Microbiol..

[23] Teng X., Zhang T., Rao C. (2025). Novel Probiotics Adsorbing and Excreting Microplastics In Vivo Show Potential Gut Health Benefits. Front. Microbiol..

[24] Zhao L., Dou Q., Chen S., Wang Y., Yang Q., Chen W., Zhang H., Du Y., Xie M. (2023). Adsorption Abilities and Mechanisms of *Lactobacillus* on Various Nanoplastics. Chemosphere.

[25] Bozkurt H.S., Yörüklü H.C., Bozkurt K., Denktaş C., Bozdoğan A., Özdemir O., Özkaya B. (2022). Biodegradation of Microplastic by Probiotic *Bifidobacterium*. Int. J. Glob. Warm..

[26] Prata J.C., da Costa J.P., Lopes I., Andrady A.L., Duarte A.C., Rocha-Santos T. (2021). A One Health Perspective of the Impacts of Microplastics on Animal, Human and Environmental Health. Sci. Total Environ..

[27] Biswas R., Debnath C., Barua R., Samanta I. (2024). Microplastics: A One Health Priority Agenda. One Health Bull..

[28] Steinmetz Z., Kintzi A., Muñoz K., Schaumann G.E. (2020). A Simple Method for the Selective Quantification of Polyethylene, Polypropylene, and Polystyrene Plastic Debris in Soil by Pyrolysis-Gas Chromatography/Mass Spectrometry. J. Anal. Appl. Pyrolysis.

[29] Fischer M., Scholz-Böttcher B.M. (2017). Simultaneous Trace Identification and Quantification of Common Types of Microplastics in Environmental Samples by Pyrolysis-Gas Chromatography–Mass Spectrometry. Environ. Sci. Technol..

[30] Krueger M.C., Harms H., Schlosser D. (2015). Prospects for Microbiological Solutions to Environmental Pollution with Plastics. Appl. Microbiol. Biotechnol..

[31] Thomas D., Schütze B., Heinze W.M., Steinmetz Z. (2020). Sample Preparation Techniques for the Analysis of Microplastics in Soil—A Review. Sustainability.

[32] Leslie H.A., van Velzen M.J.M., Brandsma S.H., Vethaak A.D., Garcia-Vallejo J.J., Lamoree M.H. (2022). Discovery and Quantification of Plastic Particle Pollution in Human Blood. Environ. Int..

[33] Schrank I., Möller J.N., Imhof H.K., Hauenstein O., Zielke F., Agarwal S., Löder M.G.J., Greiner A., Laforsch C. (2022). Microplastic Sample Purification Methods—Assessing Detrimental Effects of Purification Procedures on Specific Plastic Types. Sci. Total Environ..

[34] de Carvalho A.R., Mathieu O., Thevenot M., Amiotte-Suchet P., Bertrand X., Beugnot J.-C., Karbowiak T., Celle H. (2024). Determination of Polystyrene Microplastic in Soil by Pyrolysis—Gas Chromatography—Mass Spectrometry (Pyr-GC-MS). Anal. Lett..

[35] Lauschke T., Dierkes G., Schweyen P., Ternes T.A. (2021). Evaluation of Poly(Styrene-D5) and Poly(4-Fluorostyrene) as Internal Standards for Microplastics Quantification by Thermoanalytical Methods. J. Anal. Appl. Pyrolysis.

[36] FAO/WHO (2002). Guidelines for the Evaluation of Probiotics in Food.

[37] Leahy S.C., Higgins D.G., Fitzgerald G.F., Van Sinderen D. (2005). Getting Better with Bifidobacteria. J. Appl. Microbiol..

[38] Plessas S., Bosnea L., Alexopoulos A., Bezirtzoglou E. (2012). Potential Effects of Probiotics in Cheese and Yogurt Production: A Review. Eng. Life Sci..

[39] Brouwer H., Porbahaie M., Boeren S., Busch M., Bouwmeester H. (2024). The In Vitro Gastrointestinal Digestion-Associated Protein Corona of Polystyrene Nano- and Microplastics Increases Their Uptake by Human THP-1-Derived Macrophages. Part. Fibre Toxicol..

[40] Rui X., Fu K., Wang H., Pan T., Wang W. (2025). Formation Mechanisms of Protein Coronas on Food-Related Nanoparticles: Their Impact on Digestive System and Bioactive Compound Delivery. Foods.

[41] Kaseke T., Lujic T., Cirkovic Velickovic T. (2023). Nano- and Microplastics Migration from Plastic Food Packaging into Dairy Products: Impact on Nutrient Digestion, Absorption, and Metabolism. Foods.

[42] Casella C., Ballaz S., Luque R., Cornelli U. (2026). Chitosan with Defined Intrinsic Viscosity Enables Physicochemical Entrapment of Microplastics Under In Vitro Gastric Conditions. J. Mater. Chem. B.

[43] Geppner L., Karaca J., Wegner W., Rados M., Gutwald T., Werth P., Henjakovic M. (2023). Testing of Different Digestion Solutions on Tissue Samples and the Effects of Used Potassium Hydroxide Solution on Polystyrene Microspheres. Toxics.

[44] Kim G.-H., Jeong H., Jung I., Choi M., Kim J.-H. (2025). Functional Evaluation of Bacillus Subtilis DCP04 from Korean Fermented Soybean Paste: A Potential Probiotic Strain for Polyethylene Degradation and Adsorption. Foods.

[45] Jacquin J., Cheng J., Odobel C., Pandin C., Conan P., Pujo-Pay M., Barbe V., Meistertzheim A.-L., Ghiglione J.-F. (2019). Microbial Ecotoxicology of Marine Plastic Debris: A Review on Colonization and Biodegradation by the “Plastisphere”. Front. Microbiol..

[46] Ortiz D., Munoz M., Nieto-Sandoval J., Romera-Castillo C., de Pedro Z.M., Casas J.A. (2022). Insights into the Degradation of Microplastics by Fenton Oxidation: From Surface Modification to Mineralization. Chemosphere.

[47] McGivney E., Cederholm L., Barth A., Hakkarainen M., Hamacher-Barth E., Ogonowski M., Gorokhova E. (2020). Rapid Physicochemical Changes in Microplastic Induced by Biofilm Formation. Front. Bioeng. Biotechnol..

[48] Gao W., Xu M., Zhao W., Yang X., Xin F., Dong W., Jia H., Wu X. (2024). Microbial Degradation of (Micro)Plastics: Mechanisms, Enhancements, and Future Directions. Fermentation.

[49] Pacher-Deutsch C., Schweighofer N., Hanemaaijer M., Marut W., Žukauskaitė K., Horvath A., Stadlbauer V. (2025). The Microplastic-Crisis: Role of Bacteria in Fighting Microplastic-Effects in the Digestive System. Environ. Pollut..

[50] Dong X., Liu X., Hou Q., Wang Z. (2026). Microplastic in Milk and Dairy Products: Research Quality, Abundance, Sources, and Transfer Mechanisms. J. Hazard. Mater..

[51] Visentin E., Niero G., Benetti F., O’Donnell C., De Marchi M. (2025). Assessing Microplastic Contamination in Milk and Dairy Products. npj Sci. Food.

[52] TÜİK Milk and Dairy Product Production—December 2023—Veri Portalı—TÜİK. https://veriportali.tuik.gov.tr/tr/press/49478.

[53] Procopio A.C., Soggiu A., Urbani A., Roncada P. (2025). Interactions Between Microplastics and Microbiota in a One Health Perspective. One Health.

[54] Demarquoy J. (2025). Microplastics and Probiotics: Mechanisms of Interaction and Their Consequences for Health. AIMS Microbiol..

